# Automated speech and language markers of longitudinal changes in psychosis symptoms

**DOI:** 10.1038/s44277-025-00034-z

**Published:** 2025-06-17

**Authors:** Sunny X. Tang, Michael J. Spilka, Majnu John, Michael L. Birnbaum, Ema Saito, Sarah A. Berretta, Leily M. Behbehani, Mark Y. Liberman, Anil K. Malhotra, William Simpson, John M. Kane

**Affiliations:** 1 Northwell Health, New Hyde Park, NY USA; 2 Feinstein Institutes for Medical Research, Institute of Behavioral Science, Manhasset, NY USA; 3 Donald and Barbara Zucker School of Medicine, Department of Psychiatry, Hempstead, NY USA; 4 Linguistic Data Consortium, University of Pennsylvania, Philadelphia, PA USA; 5 Cambridge Cognition, Cambridge, UK; 6 Winterlight Labs, Toronto, Canada; 7 Department of Psychiatry, Columbia University Vagelos College of Physicians and Surgeons, New York, NY USA; 8 New York State Psychiatric Institute, New York, NY USA; 9 School of Behavioral and Brain Sciences, The University of Texas at Dallas, Richardson, TX USA; 10 Department of Psychology, Yale University, New Haven, CT USA

**Keywords:** Predictive markers, Neurological manifestations

## Abstract

We sought to evaluate the ability of automated speech and language features to longitudinally track fluctuations in the major psychosis domains: *Thought Disorder*, *Negative Symptoms*, and *Positive Symptoms*. Sixty-six participants with psychotic disorders were assessed soon after inpatient admission, at discharge, and at 3- and 6-months. Psychosis symptoms were measured with semi-structured interviews and standardized scales. Recordings were collected from paragraph reading, fluency, picture description, and open-ended tasks. Relationships between psychosis symptoms and 357 automated speech and language features were analyzed using a single component score and as individual features, using linear mixed models. We found that all three domains demonstrated significant longitudinal relationships with the single component score. *Thought Disorder* was particularly related to features describing more subordinated constructions, less efficient identification of picture elements, and decreased semantic distance between sentences. *Negative Symptoms* was related to features describing decreased speech complexity. *Positive Symptoms* domain score did not show relationships with individual features that survived p-value correction, but *Suspiciousness* was related to decreased use of nouns and *Hallucinations* was related to greater semantic distances. These relationships were largely robust to interactions with gender and race. Interactions with timepoint revealed variable relationships during different phases of illness (acute vs. stable). In summary, automated speech and language features show promise as scalable, objective markers of psychosis severity. Detailed attention to clinical setting and patient population is needed to optimize clinical translation.

## Lay summary

We used acoustic analysis and Natural Language Processing (NLP) to evaluate speech data from 66 individuals with psychosis, over time. The study identified specific language features that correlate with different psychosis symptoms as they changed over time. These insights could lead to innovative, non-invasive tools for monitoring schizophrenia and related disorders, enhancing personalized treatment approaches in psychiatry.

## Automated speech and language markers of longitudinal changes in psychosis symptoms

Take a Deep Dive into this article

## Introduction

Psychotic disorders are severe mental illnesses and include schizophrenia spectrum disorders as well as bipolar and major depressive disorders with psychotic features; the total lifetime prevalence is 2–3% [1]. While psychotic disorders are associated with significant disability, increased health care costs, family burden, and reduced life expectancy in general [2], outcomes are heterogenous and can be improved with a range of effective treatments. Antipsychotic medications remain a mainstay of pharmacologic treatment and are effective against a range of psychosis symptoms, but benefits can be limited by non-response, non-adherence, and significant side effects [3]. Psychosocial treatments like cognitive remediation, social skills training, psychotherapy, and self-management, as well as multi-disciplinary early-intervention programs have also demonstrated efficacy [4, 5]. There is a great deal of interest in developing approaches to ‘precision psychiatry’, whereby objective biomarkers can be used to facilitate early identification/diagnosis, stratify patients, optimize treatment decisions and provide patients with more effective and timely care [6].

Natural language processing (NLP) and speech and language features evaluated with automated, computerized methods may offer substantial advantages as a scalable, cost-effective, low-burden means for generating clinically relevant markers for psychosis. These methods generate a range of objective features describing the timing (e.g., latency, speaking rate), acoustic properties (e.g., frequency, amplitude), lexical characteristics (e.g., sentiment, commonness), and structure (e.g., syntax, semantic coherence, speech graph properties) of speech. They require relatively little expertise or specialized equipment to capture and, when fully developed, can be implemented in a cost- and time-efficient manner, relying on automated computer algorithms [7]. There are now multiple sources of evidence demonstrating that a range of speech and language features can be used as markers of psychosis. These methods are highly sensitive [8] and consistently predict schizophrenia diagnosis relative to healthy controls, as well as conversion to psychosis among individuals at clinical high risk [9]. Different types of speech and language features are also sensitive to different dimensions of psychosis symptoms, cognition, and functioning [10–13]; there is also indication that some categories of features can be associated with different symptom domains [14], suggesting symptom severity can be reflected in multiple aspects of speech and language.

Longitudinal studies of automated speech and language analysis in psychosis are less common and have smaller sample sizes. Girard et al. used a range of lexical, coherence, and disfluency features (e.g., features from the Linguistic Inquiry and Word Count [15], perplexity, and speech disfluencies like edits, repeats, and restarts) to longitudinally estimate psychosis symptoms in 38 participants with psychotic disorders (99 total sessions) and found promising and contrasting between- and within-participant relationships to positive and negative symptoms – with some features related to *both* positive and negative symptoms [14]. Liebenthal et al. followed 18 participants with psychotic disorder over 1 to 20 monthly timepoints (145 total interviews) used linear mixed models to demonstrate that greater conceptual disorganization was associated with increased verbosity and disfluency [16]. An earlier study by Cohen et al. included 25 participants with serious mental illness and focused on acoustic signals in a tighter time scale, with up to 5 consecutive daily assessments; there were many relationships between the vocal markers and either affective state or interactions between affective state and symptom severity, but there were no significant associations between symptoms and speech markers independent of affective state [17]. Other studies have related speech and language features to cognitive changes [18] and have examined how baseline features predict later clinical outcomes [19, 20]. In general, the scarcity of longitudinal studies in this area has been identified as a major limitation in the development of speech-based clinical applications [21].

Here, we sought to evaluate the ability of automated speech and language features to track fluctuations in psychosis symptoms among 66 participants with psychotic disorders over 160 sessions (up to 4 timepoints per participant). The long-term goal of this work is to develop a means for measuring “vital signs” in psychosis – i.e., sensitive, objective measures of psychosis severity which can be obtained rapidly and cost-effectively. We apply a broad approach, integrating information from a wide range of speech and language features assessed via several task contexts. The clinical outcomes of interest were the principal psychosis symptom domains: (1) *Thought Disorder / Disorganization*, which are early signs of both relapse and treatment response for psychosis [22, 23] and can be directly related to speech and language disturbance; (2) *Negative Symptoms*, which also include speech-related phenomena (alogia, affective flattening), can be difficult to reliably assess, and have significant implications for functional outcomes [2]; and (3) *Positive Symptoms*, which are important targets of antipsychotic treatment and predictors of hospitalization [24]. In our primary analysis, we first evaluate speech and language features in general, as a single component score, and then explore relationships with individual speech and language features to further examine what aspects of the single component score may drive any relationship with the symptom dimensions. Finally, we examine interactions with gender and race to determine the degree to which findings apply across groups. We expected that a single speech-based component score reflecting multiple aspects of speech and language would display meaningful relationships across symptom domains, and that individual speech feature relationships would vary by symptom domain. However, we did not have a priori hypotheses about the specific feature relationships given the data-driven approach employed.

## Methods

### Participants

Recruitment occurred on acute inpatient psychiatric units at The Zucker Hillside Hospital in Glen Oaks, NY. Inclusion criteria were age 15–40 years, proficient in English, current diagnosis of bipolar I disorder with psychotic features or schizophrenia spectrum disorder (schizophrenia, schizophreniform disorder, schizoaffective disorder, unspecified psychotic disorder, or brief psychotic disorder), and at least moderate positive or disorganized symptoms on admission based on the BPRS. The symptom threshold was chosen so that a larger treatment effect might be expected as there is more room for improvement; similarly, the younger age range was selected because illness course can be more dynamic in this group [5]. Individuals with substance-induced psychotic disorders were excluded, along with those with comorbidities directly affecting speech production or language ability (e.g., aphasia, stroke, autism spectrum disorder). The research procedures were approved by the institutional review board at Northwell Health, and all participants provided written consent after decisional capacity was confirmed via a consent quiz. The study was registered on ClinicalTrials.gov (NCT-05601050).

Two participant sessions were impacted by poor recording environment and therefore excluded from the analyses. A total of 66 participants and 160 sessions are described here (Table 1).

**Table 1:** Participant characteristics.

	Baseline	Discharge	3mo	6mo	p value
n	66	54	22	18	
Age (SD)	26.4 (5.3)	26.1 (4.7)	27.0 (6.0)	27.4 (5.2)	0.77
Sex (%)					0.31
Female	20 (30%)	15 (28%)	2 (9%)	2 (11%)	
Intersex	1 (2%)	1 (2%)	0 (0%)	0 (0%)	
Male	45 (68%)	38 (70%)	20 (91%)	16 (89%)	
Gender (%)					0.36
Man	45 (75%)	38 (78%)	19 (91%)	15 (88%)	
Woman	15 (25%)	11 (22%)	2 (10%)	2 (12%)	
Not Reported	6	5	1	1	
Race (%)					0.48
Asian	12 (18%)	11 (21%)	4 (18%)	3 (17%)	
Black/African American	28 (43%)	24 (45%)	11 (50%)	12 (67%)	
Other	9 (14%)	7 (13%)	0 (0%)	0 (0%)	
White/Caucasian	16 (25%)	11 (21%)	7 (32%)	3 (17%)	
Not Reported	1	1	0	0	
Ethnicity					0.60
Hispanic	10 (15%)	6 (11%)	1 (5%)	0 (0%)	
Not Hispanic	52 (79%)	45 (83%)	20 (91%)	17 (94%)	
Not Reported	4	3	1	1	
Education (SD)	14.1 (1.9)	14.0 (1.8)	14.1 (1.8)	14.0 (1.7)	0.99
Diagnosis					0.99
Bipolar w. Psychosis	4 (6%)	3 (6%)	1 (5%)	1 (6%)	
Schizoaffective	15 (23%)	13 (24%)	4 (18%)	3 (17%)	
Schizophrenia	33 (50%)	29 (54%)	11 (50%)	11 (61%)	
Schizophreniform	4 (6%)	2 (4%)	2 (9%)	0 (0%)	
Unspecified PD	10 (15%)	7 (13%)	4 (18%)	3 (17%)	
TLC Total (SD)	22.0 (14.4)	15.6 (11.3)	11.7 (12.0)	12.7 (11.4)	0.001
SANS Total Global (SD)	8.3 (3.5)	7.2 (3.4)	7.5 (3.8)	7.1 (4.9)	0.47
BPRS Positive Symptoms (SD)	20.7 (5.4)	16.7 (6.4)	14.5 (6.6)	13.5 (6.8)	<0.001

Baseline > Discharge, 3mo, 6mo. BPRS positive symptoms pairwise comparisons, Baseline > Discharge, 3mo, 6mo.

*BPRS* brief psychiatric rating scale, *SANS* scale for the assessment of negative symptoms, *SD* standard deviation, *TLC* scale for the assessment of thought language and communication, *TLC* total pairwise comparisons.

### Assessments

Participants were assessed longitudinally over 4 sessions. The first session (baseline) was conducted as soon as possible after participants were admitted. The second session (discharge) was conducted when imminent discharge was planned or within 1 week after discharge. To limit variability, a range of 1–3 weeks was imposed for the interval between the first and second sessions, reflecting average hospitalization durations and when the greatest clinical change is expected. The third and fourth sessions were conducted at 3 months and 6 months after discharge.

Diagnoses were confirmed with the SCID-IV-TR [25] using DSM-5 criteria. *Thought Disorder* was rated with the Scale for the Assessment of Thought, Language and Communication (TLC), and total score was calculated [26]. *Negative Symptoms* were rated with the Scale for the Assessment of Negative Symptoms (SANS), and global scores were totaled for the Affective Flattening, Alogia, Avolition/Apathy, and Anhedonia/Asociality domains per Robinson et al. [27, 28]. *Positive Symptoms* were rated with the Brief Psychiatric Rating Scale (BPRS) and the factor score was calculated per Overall et al. [29] totaling these items: Hostility, Suspiciousness, Uncooperativeness, Hallucinatory Behavior, Conceptual Disorganization, and Unusual Thought Content. All clinical assessments were conducted by trained assessors who underwent departmental training to establish reliability.

**Fig. 1:**
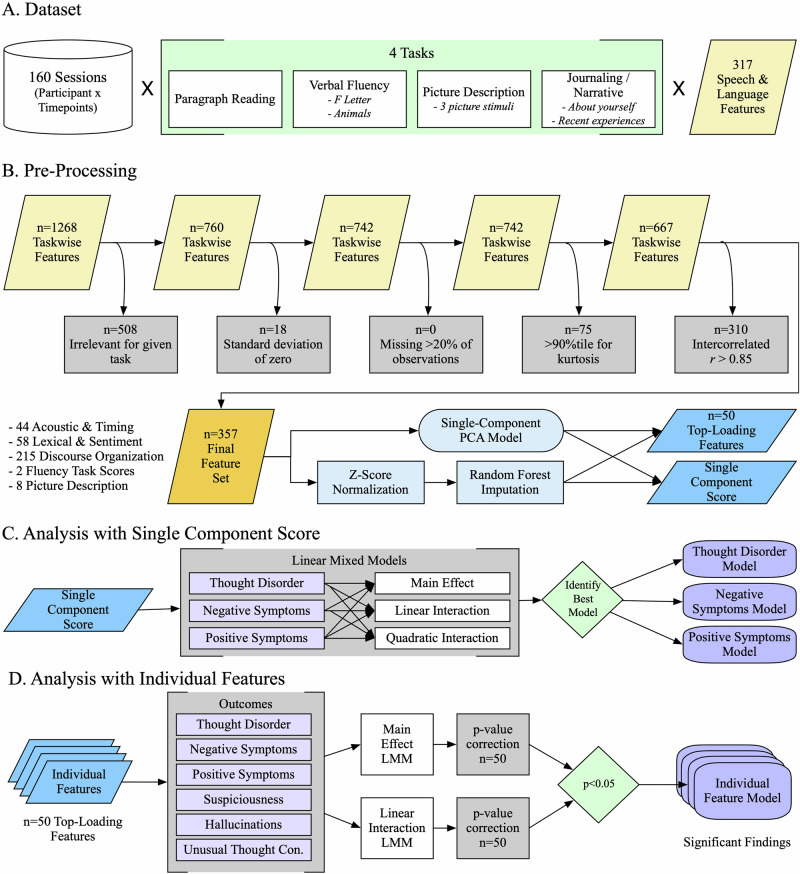
Flowchart for data processing and analyses. Schematic are shown for (**A**) Components of the dataset being analyzed, B Pre-processing steps taken for speech and language features, C Analyses based on the PCA-derived single component score, and (**D**) Analyses with individual speech and language features. PCA principal component analysis, LMM linear mixed model.

Speech was collected using iPads with the Winterlight iOS application. Participants were asked to respond to 4 verbal tasks, sometimes using multiple stimuli (Fig. 1A): paragraph reading (standardized text of 70 words, at 9th grade reading level), fluency (animal category fluency and F-letter phonemic fluency, each 1 min), picture descriptions (3 pictures per session, including scenes with characters interacting, a social conflict image derived from the Thematic Apperception Test [30], and an image from the Rorschach Test [31]), and open-ended journaling (2 self-descriptive narrative prompts: “Tell me about yourself,” and “How have you been spending your time recently?”). The full assessment took 10–15 min to complete.

### Speech and language features

Responses on the Winterlight iOS application were audio-recorded, then transcribed with a combination of automated processes and human annotation on custom software. Any speech from other speakers was identified and was removed from recordings and transcripts. The preprocessed audio and transcripts were then analyzed using the Winterlight platform (winterlightlabs.com), an automated pipeline for extracting speech and language features [32, 33]. The platform uses Python-based acoustic and NLP libraries and custom code to extract a wide range of features. Open source packages include SpaCy for parts-of-speech tagging and morphological variables [34], the Stanford NLP parser for syntactic variables [35], Praat and Parselmouth for acoustic variables [36], and GloVe and FastText models for semantic variables [37, 38]. Lexical characteristics, including sentiment and age-of-acquisition, were computed based on published norms [39–42]. In addition, sequential speech graph features [43] as well as max, min, and mean cosine similarities for adjacent word embeddings [44] were included based on promising works in these areas. Custom features were built to quantify correctly identified picture description elements. Prior to further processing, we eliminated the following features from the standard Winterlight pipeline outputs to reduce the feature set while prioritizing more interpretable features: harmonic-to-noise ratios, mel-frequency cepstral coefficients, zero-crossing rates for acoustic variables, NLTK parts-of-speech tags (which were duplicative of SpaCy tags).

For these analyses, we extracted 317 raw speech and language features for each stimulus (Fig. 1A; 45 acoustic and timing, 27 lexical characteristics, 216 discourse organization, 2 fluency task scores, 27 picture description content measures). Features were collapsed to the task-level (i.e., each task within each session was evaluated separately); where there were multiple stimuli for one task (e.g., 3 different pictures), the features were averaged across the stimuli. This produced 1,268 task-wise features (Fig. 1B). A series of features were then excluded to remove those that were not task-relevant (e.g., syntactic features removed for paragraph reading and fluency tasks), lacked sufficient variability (based on standard deviation and kurtosis), or too highly intercorrelated (Supplementary Methods; Fig. 1B). The final feature set was standardized (z-scored) and included 357 task-wise features (44 acoustic and timing, 58 lexical and sentiment characteristics, 215 discourse organization, 2 fluency task scores, 8 picture description content measures).

A single component score was calculated to represent the speech and language features globally. First, we performed a principal component analysis (PCA) on unimputed data using pairwise deletions (12 features missing up to 6 observations each), resulting in a 1-component model explaining 6.7% of the total variance. Then, we imputed missing data using random forest imputation with *missForest* v.1.5 in R. Finally, we extracted a single component score for each participant observation, represented as a z-score. The PCA and component score extraction was completed using *psych* v.2.2.5 in R. PCA was chosen over factor analysis because we wished to represent the variance from the speech and language features without an assumption about the underlying latent constructs. Feature selection through machine learning was not feasible because the sample was too small (especially at later timepoints) to set aside sufficient samples for training and testing.

Subsequently, we examined relationships between psychosis symptom domains and individual speech features to better understand specific relationships between speech features and symptom dimensions. Out of consideration for multiple comparisons, the 50 top-loading features from the PCA (described in Supplementary Table 1) were selected as candidates because they were most representative of the single component score. Notably, selecting candidate features in this way biases toward over-representation of tasks and feature types that were more common among the final feature set, i.e., picture description and journaling tasks, and discourse organization features.

### Statistical analyses

To understand how psychosis is longitudinally related to speech and language features globally, each psychosis symptoms domain (*Thought Disorder, Negative Symptoms, Positive Symptoms*) was predicted using random-intercept linear mixed models (LMMs) with the single component score and timepoint as fixed effects, and participant as the observation unit for random effects (Fig. 1C). The main effect of the component score and linear and quadratic interactions with timepoint were examined (Supplementary Table 2 details model structures). The default unstructured variance-covariance structure within nlme R package was used in all LMM analyses [45]. Timepoint was centered around the baseline, and model fit is reported with the Akaike and Bayesian Information Criteria (AIC and BIC), with greater emphasis on the BIC because it incorporates the sample size into the penalty term. Effect sizes are represented by Beta coefficients from the LMMs.

Each psychosis domain was then predicted with LMMs for the 50 top-loading individual features to better understand the contribution of specific speech and language features (Fig. 1D). Quadratic interaction models were not examined because they were not significant for any of the single component LMMs. To account for multiple comparisons with 50 features, p-values for the parameter of interest were adjusted using the Benjamini and Hochberg false-discovery rate (FDR) method [46]. Because there were no individual features for the *Positive Symptoms* domain that survived FDR correction, we hypothesized that *Positive Symptom*s may be too heterogeneous as a clinical construct and conducted post hoc analyses on individual positive symptoms. *Hallucinations*, *Suspiciousness*, and *Unusual Thought Content* were evaluated because these demonstrated sufficient variance in the sample and represent core positive symptoms.

Interactions with race and gender were explored by testing the interaction between the parameter of interest and the demographic variables. We did not examine interactions with age because the age range was relatively narrow. Education was not examined as a covariate because decreased educational attainment is a prominent *outcome* of psychosis and cannot be treated as an independent confounding variable. We also did not attempt to tease apart medication effects, but rather, approached the analyses with the goal of making inferences for *observable* psychosis symptoms, as they are. The rationale for this approach is primarily twofold: (1) this approach is analogous to standard clinical practice, where it is often not possible separate medication effects from illness manifestations; and (2) the potential effects of antipsychotics are not sufficiently well-characterized to disambiguate at this stage and in this longitudinal sample where both medication type and dosage are time-varying variables for each participant. In the existing literature, while one study found a relationship between medications with high D2 occupancy and verbal output as well as temporal features [47], another study found that the relationship between speech features and antipsychotic dose is primarily driven by symptom severity (which is in turn correlated with antipsychotic dose) [48].

All analyses were conducted in RStudio with R v.4.2.0.

## Results

### Trajectories of psychosis symptoms

**Fig. 2:**
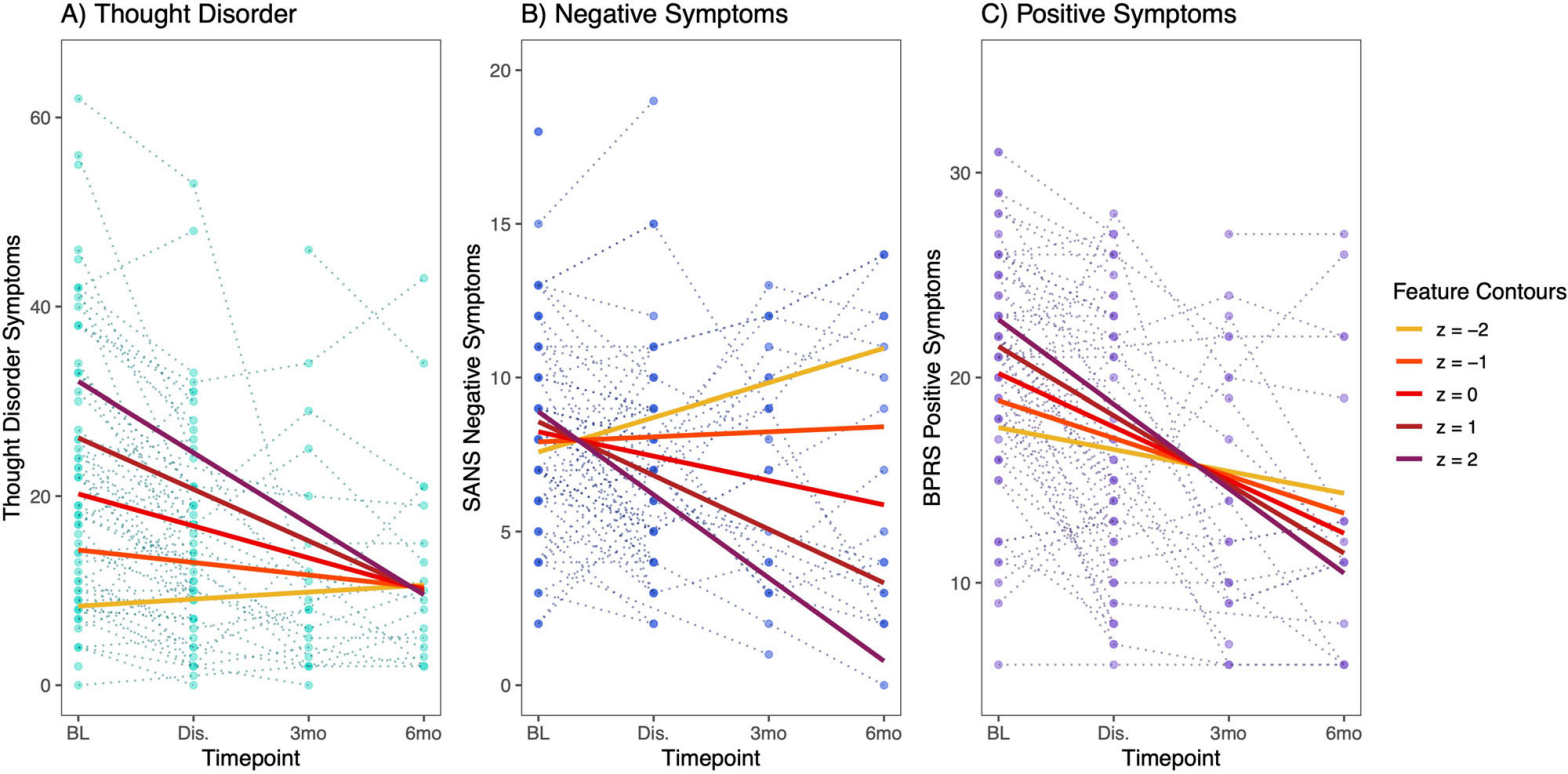
Psychosis symptoms and single component score. The single component score (“Feature”) represents the overall variance from the 357 speech and language measures in the final feature set. Variance predicted by the single component score in LMMs is shown for (**A**) *Thought Disorder* – total TLC score, (**B**) *Negative Symptoms* – total global SANS scores, and (**C**) *Positive Symptoms* – BPRS factor score. For all three symptom domains, the best fit model was the LMM including a linear interaction term between the single component score and timepoint. In each subplot, individual observations for each participant are plotted across the 4 timepoints (*Thought Disorder* – turquoise, *Negative Symptoms* – blue, *Positive Symptoms* – purple). Feature contours illustrate LMM predictions for each symptom domain at different values of the single component score (z = −2 to +2) across the 4 timepoints. When contour lines are farther apart, greater variance in symptom severity is predicted by the single component score. For example, at baseline, total TLC score (*Thought Disorder)* is estimated by the LMM to be ~30 for individuals with single component score z = 2, while total TLC score is estimated at ~10 for individuals with single component score z = −2. At 6mo follow-up, there is very little difference in the estimated *Thought Disorder* severity regardless of the single component score. The opposite pattern can be observed for *Negative Symptoms*, for which little variance is explained at baseline and more variance is explained at later timepoints, though in the opposite direction from *Thought Disorder*. For *Positive Symptoms*, the single component score predicts an opposite pattern during the inpatient timepoints compared to the two follow-ups.

Changes in psychosis symptoms followed expected patterns (Table 1), with generally declining symptoms and significant overall effect of timepoint for *Thought Disorder* (*p* = 0.001) and *Positive Symptoms* (*p* < 0.001). There was no overall effect of timepoint on *Negative Symptoms* (*p* = 0.47). However, there was significant individual variability, as can be observed from the individual datapoints plotted in Fig. 2. Pairwise comparisons showed that both *Thought Disorder* and *Positive Symptoms* were elevated at Baseline compared to each of the following timepoints, with no significant differences among the latter 3 timepoints. There was significant attrition across timepoints, but demographic characteristics and diagnoses did not significantly differ across timepoints (Table 1). Baseline clinical ratings also did not differ among participants who were able to return for different numbers of timepoints (Supplementary Table 3). Therefore, the observed clinical trajectories appear to be largely representative of the group as a whole, despite incomplete data at later timepoints.

### Psychosis domains and single component score

There were significant longitudinal relationships between the single component score and all three psychosis symptoms domains (Table 2). For *Thought Disorder*, the relationship with the single component score was significant in all three models (main effect, linear interaction, and quadratic interaction models; refer to Supplementary Table 2 for model structure details, and Supplementary Table 4 for unabbreviated results). The linear interaction model was chosen as the best fit for *Thought Disorder* based on the BIC model performance statistic, suggesting that this model explained the most variance in symptoms. In this model, both the main effect for the single component score and the linear interaction term for timepoint were significant – suggesting that there was a time-varying relationship superimposed on a non-time-varying relationship. As reflected in Fig. 2A, higher values on the single component score predicted higher *Thought Disorder* symptoms, with more variance explained at the initial timepoints than at the later ones. For *Negative Symptoms* and *Positive Symptoms*, only the linear interaction term was significant, and the linear interaction models were chosen as the best fit in both cases (suggesting a time-varying relationship between the single component score and symptom severity). As shown in Fig. 2B, in contrast to *Thought Disorder, lower* values on the single component score were related to *greater Negative Symptoms*, and the greatest variance is predicted at 3- and 6-month follow-up. For *Positive Symptoms*, the polarity of the relationship reverses after discharge (Fig. 2C).

**Table 2:** Linear Mixed Models Results.

		Int.	Timept.	Main Effect			Lin. Interaction			Model	
Feature	Model	Coeff.	Coeff.	Coeff.	p	p-adj.	Coeff.	p	p-adj.	AIC	BIC
A. Features Relating to Thought Disorder											
Single component score	L	20.2	−3.4	5.9	<0.001		−2.1	<0.001		1212	1230
Subordinating conjunctions (JOU)	M	20.6	−3.3	4.2	<0.001	<0.001				1212	1227
Min. utterance semantic dist. (Google) (JOU)	M	20.8	−3.5	−3.5	<0.001	0.001				1216	1231
Min. utterance semantic distance (fastText) (PIC)	M	20.9	−3.7	−3.3	<0.001	0.002				1218	1233
Picture units identified:Action (PIC)	M	21.6	−4.3	−3.6	<0.001	0.002				1218	1234
Total audio duration (JOU)	M	20.8	−3.5	3.5	<0.001	0.002				1219	1234
Picture units identified (PIC)	L	21.0	−3.8	−5.6	<0.001		2.2	<0.001	0.03	1214	1232
Noun phrase: Noun (JOU)	L	21.1	−4	−4.3	<0.001		2.3	0.002	0.03	1219	1238
B. Features Relating to Negative Symptoms											
Single component score	L	8.2	−0.8	0.3	0.38		−1.0	<0.001		844	862
Subordinate clause: preposition + sentence (JOU)	L	8.2	−0.6	1.1	0.003		−0.9	<0.001	0.02	850	868
Min. utterance semantic distance (fastText) (PIC)	L	8.2	−0.6	−0.4	0.2		0.7	0.001	0.02	851	870
Imageability (PIC)	L	8.3	−0.5	−0.5	0.2		0.7	0.002	0.02	852	870
Age of acquisition (PIC)	L	8.1	−0.4	0.6	0.1		−0.7	0.002	0.02	853	871
Adjective phrase length (PIC)	L	8.3	−0.7	0.1	0.7		−0.9	0.003	0.02	847	865
Average utterance semantic distance (Google) (PIC)	L	8.2	−0.6	−0.1	0.8		0.6	0.003	0.02	850	868
Number of edges (JOU)	L	8.1	−0.5	−0.7	0.05		0.9	0.004	0.02	854	872
C. Features Relating to Positive Symptoms											
Single component score	L	20.2	−2.6	1.3	0.03		−0.8	0.04		997	1015
D. Features Relating to Suspiciousness											
Subordinating conjunctions (JOU)	M	3.9	−0.5	0.5	0.001	0.03				663	678
Noun phrase rate (JOU)	M	3.9	−0.5	−0.5	0.002	0.03				663	678
Noun phrase: Noun (JOU)	M	3.9	−0.5	−0.5	0.002	0.03				663	678
Imageability (JOU)	M	3.9	−0.4	−0.5	0.003	0.04				664	680
Number of edges (JOU)	M	3.9	−0.5	−0.4	0.005	0.05				665	680
E. Features Relating to Hallucinations											
Age of acquisition: nouns (JOU)	M	3.6	−0.5	−0.5	0.001	0.06				645	660
Min. utterance semantic distance (fastText) (PIC)	M	3.5	−0.5	0.5	0.003	0.06				646	662
Average utterance semantic distance (Google) (PIC)	M	3.5	−0.4	0.5	0.003	0.06				646	662

See Supplementary Table 4 for unabbreviated LMM results, including quadratic interaction models. Given the space constraints, we selected the most significant findings for inclusion in Table 2, as well as additional individual features that best represented the range of all significant findings.

*M* main effect, *L* linear interaction effect, *JOU* journaling task, *PIC* Picture description task.

### Psychosis symptoms and individual speech and language features

Fifty speech and language features were evaluated individually for their longitudinal relationships with the psychosis symptoms, with many significant even after correcting for multiple comparisons (Table 2; unabbreviated results – Supplementary Table 4).

**Fig. 3:**
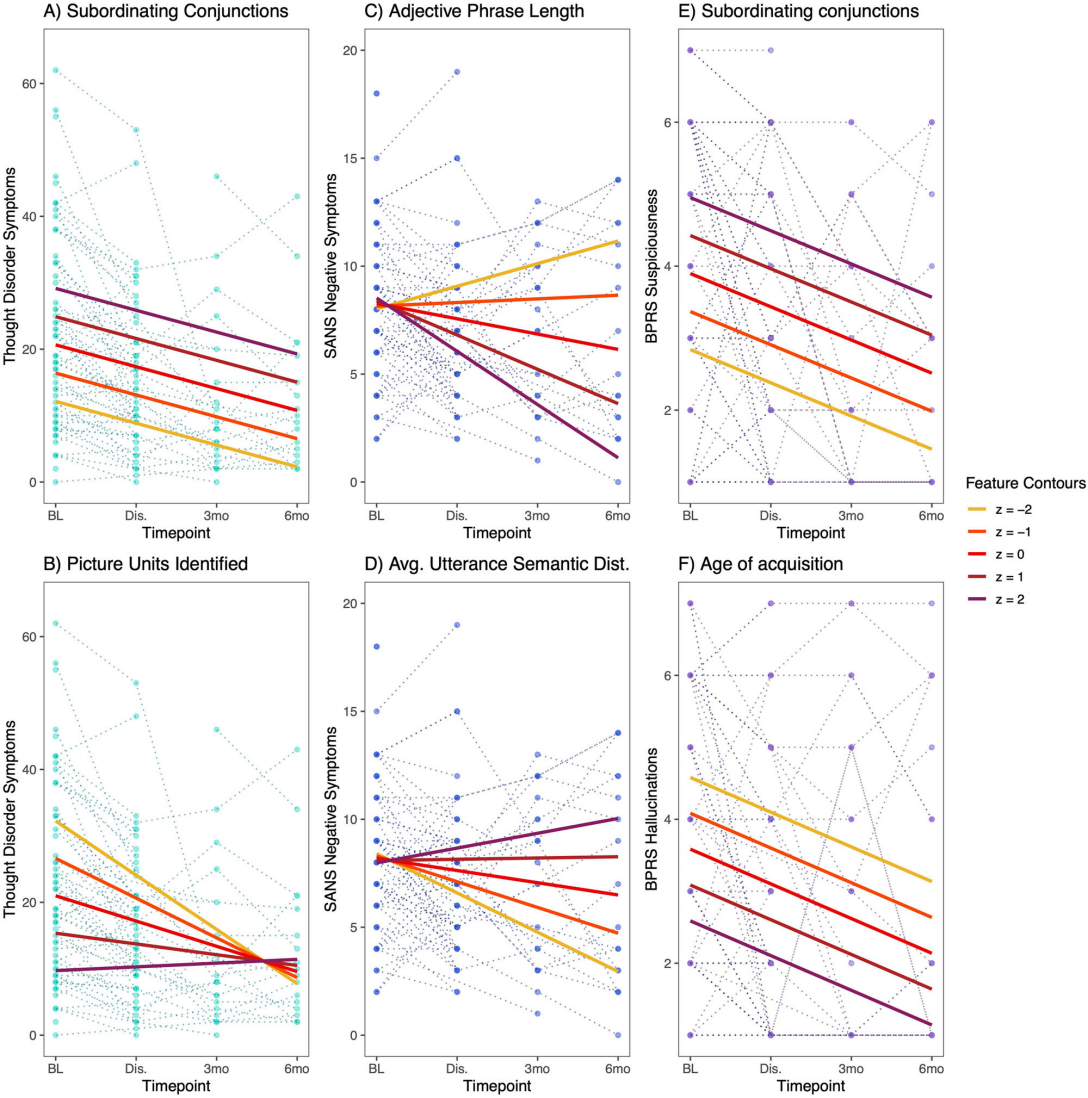
Psychosis symptoms and individual speech and language features. Illustrative examples are shown for relationships between individual features and psychosis symptoms. Individual observations are plotted across the 4 timepoints (*Thought Disorder* – turquoise, *Negative Symptoms* – blue, *Positive Symptoms* – purple). Feature contours illustrate LMM predictions for each symptom domain at different values of the feature score (z = −2 to +2). In panels (**A**, **E**), we observe main effects with higher feature scores being related to higher symptoms at all timepoints; the reverse is true in panel (**F**). Linear interactions with timepoint are evident in panels (**C**, **D**) where these features explain little variance at baseline and increasing amounts of variance at later timepoints. Panel (**B**) also shows a linear interaction in the opposite direction, with greater variance explained in-hospital than at the follow-ups.

*Thought Disorder* was significantly related to 15 features: 12 through main effects, and 3 through linear interactions with timepoint. These prominently included subordinate sentence constructions with more subordinate clauses reflecting greater *Thought Disorder* (7 related features, e.g., Fig. 3A). Lower minimum semantic distance between adjacent sentences (i.e., closer in meaning) and fewer entities correctly identified on picture description tasks (e.g., Fig. 3B) were also related to greater *Thought Disorder*.

*Negative Symptoms* was significantly related to 14 features, all through linear interactions with timepoint. There was a general pattern of higher *Negative Symptoms* being related to features describing decreased speech complexity: words that are more easily visualized (imageability), less modifiers through adjectives and adverbs (e.g., Fig. 3C), fewer connections (number of edges on speech graphs), and greater jumps in content (less elaboration, higher semantic distance between sentences; e.g., Fig. 3D). As was true for the single component score, the individual features accounted for greater variance in negative symptoms at follow-up.

None of the features were significantly related to *Positive Symptoms* after correction for multiple comparisons. Due to the heterogeneity of the domain, and to better understand the initially significant relationship between *Positive Symptoms* and the single component score, we examined the individual items for *Suspiciousness*, *Hallucinations*, and *Unusual Thought Content*. *Suspiciousness* was significantly related to 5 features, all through main effects. Higher *Suspiciousness* appears to be related to more subordinating conjunctions (e.g., Fig. 3E) and less use of nouns in the journaling task. *Hallucinations* demonstrated 3 trend-level relationships which were included for illustrative purposes, including relationships between *Hallucinations* and using words with lower age of acquisition (learned at earlier age; Fig. 3F) and greater semantic distances. There were no significant relationships between speech and language features and *Unusual Thought Content*.

### Interactions with gender and race

Interactions between the speech and language parameter of interest and demographic variables were examined for the single component score and for the individual features highlighted as illustrative examples in Fig. 3 (model structure detailed in Supplementary Table 2). There were no significant interactions between gender and any of the speech and language features (though there were main effects, they did not impact the relationship with speech features). For race, there were significant interactions with the single component score in *Negative Symptoms* and *Positive Symptoms*, and also with average utterance semantic distance in *Negative Symptoms* (Supplementary Table 5). The effect of race appeared to be primarily driven by a difference between White/Caucasian participants and all other groups, with highly divergent patterns (Supplementary Fig. 1). There were no interactions for race and any of the features evaluated for *Thought Disorder, Suspiciousness*, or *Hallucinations*, nor for the other individual features predicting *Negative* and *Positive Symptoms*.

## Discussion

In this study, we used speech and language features derived from automated analyses to infer concurrent psychosis symptoms severities across four timepoints, as participants were assessed during and after acute psychiatric hospitalizations. We examined changes in thought disorder, negative symptoms, and positive symptoms as measured by assessor-rated clinical scales, expecting that thought disorder and positive symptoms would diminish substantially because these symptom domains are most responsive to antipsychotic treatments in the acute care setting [49], and expecting little change in negative symptoms, because they are relatively treatment refractory [50]. The results confirmed our expectations regarding overall trends across the sample, but considerable individual variability in symptoms trajectories could also be observed, some in opposite direction – thus highlighting the importance of being able to detect symptoms trajectories on the *individual* level. This is one of only a handful of studies to examine objective speech and language features and fluctuations in psychosis symptoms in a longitudinal manner [14, 16, 17].

Our primary finding was that each of the major domains of psychosis symptoms was longitudinally related to speech and language features, on a global level. That is, taking a single component score representing 357 features, we found that objective speech and language features were related to psychosis symptoms across four timepoints. This was true for *Thought Disorder*, *Negative Symptoms*, and *Positive Symptoms*. However, different patterns in the relationships could be observed – for example, with higher component scores being related to higher *Thought Disorder* but decreased *Negative Symptoms*. The single component score captured a relatively small proportion of the variance in the speech and language features. This was expected, and likely due to the large number of features included and the numerous aspects of speech and language that can be identified on a conceptual level. It makes sense that we would need multiple and perhaps numerous measures to capture a large proportion of variance in the way a person speaks. Our use of the single component score in this analysis was intended to illustrate the *potential* of using a combination of speech and language features to infer changes in psychosis symptoms while under the constraints of the current sample. Building a machine learning model, for example, with cross-validation, feature selection, and an independent validation sample would have been ideal but was not feasible here. Likely, as the field continues to build on this work, we will discover the best mix of features to describe each symptom dimension of importance.

Next, we examined individual features to better understand how psychosis symptoms are related to specific measures represented in the single component score and found many significant relationships, particularly for *Thought Disorder* and *Negative Symptoms*. The 50 top-loading individual features were explored in order to scale this exploratory and descriptive analysis in proportion to the sample size available, with attention to limiting Type I errors (from multiple comparisons) and Type II errors (from rejecting noteworthy findings due to correcting p-values for too many comparisons). Some features were related to multiple symptom areas (e.g., subordinating conjunctions, semantic coherence, imageability), though sometimes through different patterns, while other features were unique to one symptom domain. It seems promising that, if confirmed and better understood in future studies, different kinds of speech and language measures may be combined to provide specificity for different kinds of psychosis symptoms. In fact, our findings suggest individual positive symptoms may be better modeled by speech features than the more heterogeneous construct of the *Positive Symptoms* domain.

When examining individual speech and language features, we found potentially interpretable patterns in their relationships with the psychosis symptom domains. *Thought Disorder* was related to several features reflecting more subordinated constructions (structures adding on additional information to the ongoing sentence) and less efficient identification of entities in picture descriptions. Though further work is needed to fully understand the connections between behavior and derived features, this might be interpreted as reflecting speech where ideas are (excessively) layered on top of one another, failing to communicate purposeful content. For example, one participant produced the following description during the picture description task (subordinated phrases in italics): “It might be like dusk *or something like that*. It it is dusk *or something like that*. It might be in the morning but not likely. Well, it could be in the morning *because you know mice and stuff*. They’re usually active at night and into the morning or so so yeah and the car is outside.” *Thought Disorder* was also related to decreased minimum distances in the semantic content of adjacent sentences, which would support the proposal of a ‘shrinking semantic space’ with shorter distances between concepts [51]. *Negative Symptoms* on the other hand were related to several features describing a reduction in complexity on a word-choice level (higher imageability), syntactic level (fewer adjectives and adverbs), and discourse organization level (fewer edges connecting speech graphs). *Negative Symptoms* were additionally related to higher average semantic distance between adjacent sentences, particularly during the picture description task, which may reflect less elaboration and moving on to the next picture element instead of providing additional detail. Altogether, this fits with earlier work that links psychosis with decreased “semantic density” [52] and “idea density” [53]. Both *Thought Disorder* and *Negative Symptoms* are important clinical targets for automated assessment, and strongly related to functional outcomes [54, 55]. In addition, conceptual disorganization is related to non-response to antipsychotic medications [56], is among the early signs of psychosis relapse [23], and is responsive to successful antipsychotic and psychosocial treatment [22, 49].

The *Positive Symptoms* domain appeared to demonstrate the least impressive findings for the single component score (significant but weaker relationship, visualized with less separation across the contour lines in Fig. 2C), and for the individual features, with no feature surviving correction for the 50 comparisons. We recognize that positive symptoms are major treatment targets [57], and are therefore important areas for speech and language features to accurately reflect. When exploring relationships with individual features, we found stronger relationships with *Suspiciousness* and *Hallucinations* as individual symptoms, compared to *Positive Symptoms* as a domain. *Unusual Thought Content* did not demonstrate relationships with speech and language features. These findings underscore the need to model symptoms at the correct level of precision, as different psychosis symptoms may have contrasting or even conflicting relationships with speech and language features. *Positive Symptoms* as a domain score, as well as the BPRS item *Unusual Thought Content*, may be too heterogeneous to identify meaningful speech and language correlates.

Some relationships between symptom domains and speech features were less readily interpretable. For example, where *Thought Disorder* and *Suspiciousness* were related to the proportion of noun phrases consisting of a singular noun, or where *Negative Symptoms* were related to subordinating clause productions consisting of a preposition and sentence. At this stage, it is unclear whether these are true reflections of the underlying psychosis constructs (perhaps by reflecting a certain pattern of expression), or if they are artifacts of this study design or an unknown confound. By taking a more inclusive, exploratory approach, it opens the possibility of finding unlooked for patterns that may prove to be informative, if they can be replicated in subsequent studies. At this stage, these findings should be considered an initial signal of interest for further exploration. Of note, because inter-correlated features were excluded from the analysis to improve interpretability and computational efficiency, other individual items may be of clinical importance but may not be reflected in the findings for individual features.

The effect of timepoint on the relationships between features and symptoms is worth noting. Many of the highlighted models included a linear interaction between timepoint and feature, implying that the information provided by the speech and language features had different implications for symptom severity depending on the timing of the assessment – whether in a hospitalized acute setting, or after stabilization and discharge. This pattern is clearest for *Negative Symptoms*, where the single component score and all of the individual features demonstrated a pattern where very little variance is predicted during the first 2 timepoints, and greater variance is accounted for during follow-up. A plausible explanation is that *Negative Symptoms* may be masked by more prominent *Positive Symptoms* and disorganization during acute psychosis exacerbations, as well as being superimposed upon sedation and medication side effects in the hospitalization setting. For *Thought Disorder* and *Positive Symptoms*, the single component score was described by a linear interaction with timepoint, but the majority of individual comparisons showed a main effect of the speech and language feature – i.e., variation in the speech feature had a consistent effect on the predicted symptom severity across timepoints, superimposed on an overall expectation of declining symptoms. Overall, these results suggest that phases of illness may affect some relationships between speech and psychosis symptoms, while others remain consistent. To our knowledge, this has not been previously examined, as most previous studies have focused on cross-sectional relationships.

In most cases, the relationships between psychosis symptoms and speech and language features were robust to the effects of gender and race. However, there were exceptions for race. It is unclear whether these were reproducible effects, or if these findings are driven by the relatively small sample of White/Caucasian participants. We are unaware of such demographic interactions having been previously tested or reported. They were explored here because we felt it was important early in the development of these potential clinical markers to be aware of the potential for bias or differential accuracy in different groups.

Many questions remain unanswered by the present study. While this is the largest longitudinal study of computational speech and language features and psychosis, to our knowledge, this study was not adequately powered to address some important concerns. We experienced a decline in participation for the later timepoints especially, which we attribute to the disruptive nature of an acute hospitalization event and pandemic-related considerations during the data collection period. Importantly, we focused on concurrent inference of psychosis symptom severity with the speech and language features and did not predict outcomes in a prospective manner. The results also prompt us to question the appropriate granularity at which clinical constructs should be investigated. While the improved prediction of individual *Positive Symptoms* items over the global *Positive Symptoms* score would suggest higher accuracy with more detailed clinical targets, an over-specification may also increase sensitivity to assessment environment and individual participant variability. Perhaps different approaches will be optimal for different clinical applications. In contrast to our approach of combining and exploring many different analytical strategies for evaluating speech and language, including acoustic, temporal, syntactic, and semantic properties [58], others have approached the shared goal of identifying psychosis-related speech and language measures through a narrower focus driven by specific hypotheses. For example, recent evidence has been mounting in support of a “shrinking semantic space” being a prominent marker of language disturbance in schizophrenia [51]. However, we prioritized a more inclusive, hypothesis-generating approach here for feature selection, which we feel is complementary to these hypothesis-driven studies, especially at a stage when existing publications have been limited in sample size as well as the range of features examined. Features were also treated in a task-wise manner to allow for variability in the effects being elicited by different tasks; further exploration focusing on how tasks affect feature variance and relevance to psychosis symptoms is warranted. We took the approach of examining observable symptoms regardless of medication effects; while this is a practical reflection of how such an approach may be implemented, we must consider that the effects observed here may be exaggerated or muted by interactions with medications.

The clinical implications are substantial for developing a scalable, cost-effective, low patient burden method of obtaining objective measurements of psychosis severity; this is the eventual goal of this line of research. Rehospitalizations are a major driver of poor outcomes in psychosis [24]. A sensitive, efficient tool can be used to monitor patients for exacerbations between visits, and alert clinicians to intervene in a timely manner. Medication adherence is similarly critical in psychosis management, with side effects being a common reason for discontinuation [59]. Speech and language biomarkers could potentially be used for more accurate and faster titration to the optimal dose and medication type, thereby decreasing patient distress, minimizing side effects, and improving adherence. Differential diagnosis also remains a challenge in some community and primary care settings [60], and could be aided by an objective biomarker to guide decision-making. Critically, due to the difficulty of demonstrating effectiveness for novel psychotherapeutics, there has been a slowing down of pharmaceutical investment in psychiatric disorders [61]. Speech and language markers of psychosis severity can serve as objective outcome measures and facilitate the discovery and approval of novel effective pharmacologic and psychosocial treatments. The current work is an initial step toward these worthwhile goals. Much needs to be completed to make these goals a reality, including but not limited to determining the most efficient task or tasks for evaluation in each clinical context, elucidating the contribution of medications to observed relationships, and reaching for a more thorough understanding of how individual features reflect clinical symptoms concurrently and predictively.

In total, our findings support the use of automated speech and language features as objective markers for tracking psychosis symptoms severity. Different types of psychosis symptoms appear to be distinguishable with different speech and language measures. The present study is a critical initial step in deploying speech biomarkers for psychosis in a longitudinal context.

### Citation diversity statement

The authors have attested that they made efforts to be mindful of diversity in selecting the citations used in this article.

## Supplementary information

Supplemental Materials

## Data Availability

Code from data analyses are available via Github (https://github.com/sunnyxtang/LPOP). Deidentified data are available upon reasonable request to the authors.
